# A body-fixed-sensor-based analysis of stair ascent and sit-to-stand to detect age-related differences in leg-extensor power

**DOI:** 10.1371/journal.pone.0210653

**Published:** 2019-01-17

**Authors:** Evelien Van Roie, Stijn Van Driessche, Bas Huijben, Remco Baggen, Rob C. van Lummel, Christophe Delecluse

**Affiliations:** 1 Department of Movement Sciences, Physical Activity, Sports and Health Research Group, KU Leuven, Leuven, Belgium; 2 McRoberts BV, The Hague, The Netherlands; University of L'Aquila, ITALY

## Abstract

Human ageing is accompanied by a progressive decline in leg-extensor power (LEP). LEP is typically measured with specialized and expensive equipment, which limits the large-scale applicability. Previously, sensor-based trunk kinematics have been used to estimate the vertical power required to elevate the body’s center of mass during functional tests, but the link with LEP and age remains to be investigated. Therefore, we investigated whether a body-fixed sensor-based analysis of power during stair ascent (SA) and sit-to-stand (STS) is positively related to LEP and whether its ability to detect age-related declines is similar. In addition, the effect of load during SA and STS was investigated. 98 adults (20–70 years) performed a leg press to assess LEP, SA and 5-repetition STS tests. In SA and STS, two conditions were tested: unloaded and loaded (+10% body mass). An inertial measurement unit was used to analyze (sub)-durations and vertical power. SA and STS power were more related to LEP than duration parameters (i.e. 0.80–0.81 for power and -0.41 –-0.66 for duration parameters, p < 0.05). The average annual age-related percent change was higher in SA power (-1.38%) than in LEP (-0.86%) and STS power (-0.38%) (p < 0.05). Age explained 29% in SA power (p < 0.001), as opposed to 14% in LEP (p < 0.001) and a non-significant 2% in STS power (p = 0.102). The addition of 10% load did not influence the age-related decline of SA and STS power nor the relationship with LEP. These results demonstrate the potential of SA tests to detect age-related deterioration in neuromuscular function. SA seems more sensitive to detect age-related changes than LEP, probably because of the additional balance component and plantar- and dorsiflexor activity. On the contrary, STS is less sensitive to age-related changes because of a ceiling effect in well-functioning adults.

## Introduction

Human ageing is accompanied by a progressive decline in neuromuscular function [1]. Together with age-related changes that occur in muscle morphology and architecture [2, 3], this decline is characterized by substantial reductions in muscle strength, muscle power and rapid force production [4–7]. Age-related declines in power and rapid force production have been shown to precede and to be more pronounced than the decline in muscle strength [7–9]. This greater decline can be attributed to a combination of age-related changes, such as a decreased maximum shortening velocity, a preferential denervation and atrophy of type II fibers, and declined neural activation [10–12]. In addition, muscle power appears to be a better predictor of functional capacity than muscle strength per se [13, 14]. Consequently, the evaluation of muscle power has been suggested as a sensitive method for the detection of early deterioration of neuromuscular function.

Leg-extensor power is typically measured with specialized and expensive exercise equipment that requires skilled lab personnel (e.g., pneumatic or isotonic resistance machines, force plate analysis, Nottingham Power Rig) [15–17] and is often limited to single joint movements (e.g., isokinetic dynamometers) [7, 18]. Most of these modalities eliminate the balance component of performance and might therefore not be able to fully capture leg-extensor power from a functional perspective. In addition, large-scale applicability of these methods in clinical settings is limited.

Clinically feasible measurements of leg-extensor power should be easy to administer, limited in time, low cost, functionally relevant and suitable for both community-dwelling and institutionalized elderly. In this regard, simple field tests, such as stair ascent and 5-repetition sit-to-stand tests, are preferable. In addition to accurate timing, body-fixed sensors can provide additional information on sub-phase durations and kinematic properties of a functional movement [19]. Previously, researchers have used sensor-based trunk kinematics to estimate the vertical power required to elevate the body’s center of mass during a transfer from sit to stand [20, 21]. A similar approach can be used during stair ascent. To date, no research has investigated the effects of age on sensor-based stair ascent and sit-to-stand power nor compared it to the effects of age on leg-extensor power.

It should be noted that both rising from a chair and stair ascent might in some ways represent a ‘controlled’ effort that does not allow for the full potentiation of power in the concentric phase of the movement [22], especially in younger individuals. This results in a ‘ceiling’ effect, which means that performance is not measurable above a certain level. Performance declines as a consequence of ageing might therefore be underestimated, limiting the potential of the test to detect early deterioration of neuromuscular function. Increasing the load, for instance by wearing a weighted vest, might be a way to increase the functional demand and force/power output needed to complete the task [23–26]. In that way, loaded stair ascent or sit-to-stand tests might be more sensitive than unloaded tests to detect early deficits in leg-extensor neuromuscular function.

Therefore, the aim of the current study was to investigate whether power during stair ascent and sit-to-stand is a good representation of leg-extensor power in a healthy community-dwelling population. In addition, its sensitivity to detect early age-related changes was examined and compared to the sensitivity of leg-extensor power. In attempt to overcome the potential ceiling effect in stair ascent and sit-to-stand, the effect of an additional external load on the relationship with leg-extensor power and on the sensitivity to detect age-related differences was investigated.

## Materials and methods

### Subjects

Subjects aged 20 to 70 years (n = 8–11 ♂ and n = 9–10 ♀ per decade) were recruited through local advertisements and oral communications. Exclusion criteria were unstable cardiovascular disease, recent surgery, neuromuscular disease, infection or fever and pregnancy. Subjects were recreationally active, with no systematic engagement in strength or endurance training. In total, 97 subjects (n = 49 ♂ and n = 48 ♀) provided written informed consent and were included in the analyses. Recruitment and testing took place from February until July 2016. The study was approved by the Medical Ethics Committee UZ KU Leuven / Research in accordance with the declaration of Helsinki.

### Outcome measurements

#### Leg-extensor power

Multi-joint leg-extensor power was measured with an isokinetic leg press device. The technical details and procedures have been previously described, but are repeated here for clarity [17]. Measurements were performed unilaterally on the right side. Subjects were seated on a backward-inclined (5°) chair, which was vertically and horizontally adjustable. The lever arm was adjustable to allow standardization of the multi-joint range of motion. Range of motion was set from a knee joint angle of 90° to 160° and from a hip joint angle of 70° to 115°, with a fully extended leg corresponding to a knee and hip angle of 180°. The hips and shoulders were stabilized with safety belts. The right foot was fully supported and fixed to the foot plate using a solid strap with the lateral malleolus aligned with the point of force application. Subjects wore flat non-cushioning shoes to minimize the cushioning effect during contractions. The angular velocity of the lever arm was fixed at 420°/s. Three maximal knee extension tests were performed. Subjects were instructed to push as fast and as hard as possible over the full range of motion.

Signal processing. Torque and velocity signals were captured at 1000 samples/s and processed offline using Matlab (The Mathworks Natick, USA). Torque signals were filtered using a fourth-order low-pass Butterworth filter with a 20Hz cut-off frequency. Instantaneous power (watt) was calculated as the product of torque (Nm) and velocity (rad/s) throughout the movement. Leg-extensor power was identified as the highest value of the power-time curve (LEP, watt). Intra-rater reliability of LEP in our lab was excellent, with ICC of 0.96 and CV(%) of 6.1.

#### Functional performance tests

For the stair ascent test (SA), subjects were instructed to ascend a flight of 6 stairs as fast as possible without using the handrail. The dimensions of the steps of the stair were 18cm height and 26.5cm depth (with 4.5cm sticking out). For the 5-repetition sit-to-stand test (STS), subjects were instructed to perform five sit-to-stand cycles as fast as possible with both arms crossed over the chest, in accordance with the STS protocol of the Short Physical Performance Battery [27]. A standard chair (0.46m height and 0.42m depth) without arm rests was used.

Both functional performance tests were performed four times, i.e. twice unloaded and twice loaded using a weighted vest with 10% body mass. The added external load was introduced to overcome the potential ceiling effect in both STS and SA performance. A weighted vest was chosen to add central loading, as previous research demonstrated that central loading in STS tests increases knee-extensor muscle activity [25]. The best result for each of the parameters explained below was used in the analyses. During the tests, subjects wore a portable, small and light-weighted inertial sensor that integrates a triaxial accelerometer and a triaxial gyroscope (DynaPort MoveTest, McRoberts BV, The Hague, The Netherlands). The sensor was inserted in an elastic belt and positioned on the lower back at the lumbar vertebra. Sampling rate was 100 samples/s. Data were analyzed using commercially available software (DynaPort MoveTest, McRoberts, The Hague, The Netherlands).

Signal processing. The measurement of 3-dimensional accelerations and angular velocities of the trunk allowed a detailed analysis of the SA and STS movement. This method has been described for STS in more detail elsewhere [28, 29]. Vertical velocity (v_VT_, m/s) was calculated by integration of the vertical acceleration (a_VT_, m/s²) signal. Instantaneous power (watt) was calculated by means of the following formula: power = BM x (a_VT_ + 9.81 m/s²) x v_VT_ x cos(θ), with BM as body mass in kg (+ additional external load) and θ defined as the angle between the vertical velocity and vertical force vector, which can either be 0 or 180 degrees.

Stair ascent was divided in six ascending steps. The start of an ascending step was defined as the point where the vertical velocity exceeded 0.1 m/s or when a local minimum greater than 0.1 m/s was found in the vertical velocity signal. A step ended when the next step was initiated (i.e., velocity exceeded 0.1 m/s after a drop below that cut-off or at a local minimum in the velocity signal greater than 0.1 m/s) or when the vertical velocity dropped below 0.1 m/s for the last step. Each step was divided in two phases based on the vertical velocity: a rise phase (v_VT_ > 0.1 m/s, vertical displacement) and a support phase (v_VT_ < 0.1 m/s, no vertical displacement). Total ascent duration (s) was calculated as the sum of the durations of the six steps. Ascent rise duration (s) was calculated as the sum of the six rise durations. Mean power (watt) was calculated for each single rise phase. The repetition with the highest mean power output (for power parameters) and the trial with the lowest duration (for duration parameters) were used in the analyses. Test-retest reliability of SA duration and power parameters is an important issue considering that individuals might alter their movement pattern or speed in separate test sessions, resulting in changes in the measured parameters. Reliability in our lab was excellent, with ICC of 0.93–0.94 and CV(%) of 4.0–6.3.

In the STS, five STS cycles with sub-phases (sit, sit-to-stand transition, stand, stand-to-sit transition) were defined according to previous procedures [19]. Total STS duration was calculated from the start of the movement until the fifth repetition ended in a standing position [27]. Sit-to-stand transition duration was calculated as the sum of the durations of the 5 sit-to-stand transitions. Mean power (watt) was calculated for each single sit-to-stand transition and the highest value for each of the parameters was used in the analyses. Reliability of the instrumented STS test has been demonstrated before in a geriatric population [30].

**Fig 1:**
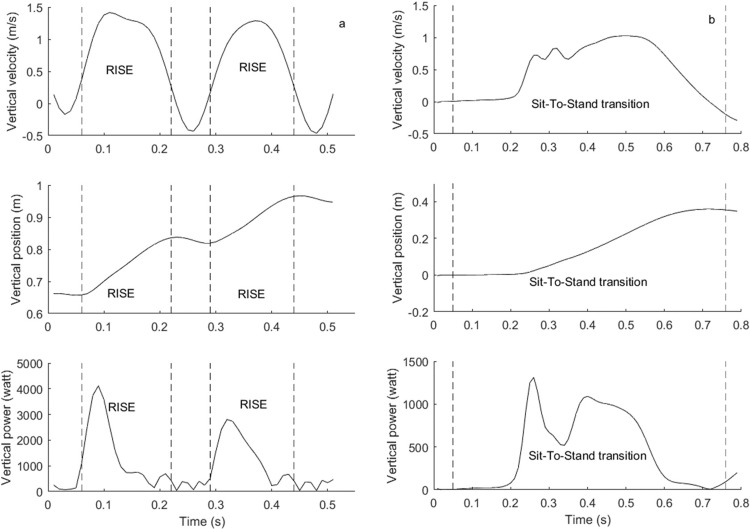
**Graphical visualization of the sensor-based analyses of vertical velocity, position and power in stair ascent (a) and sit-to-stand (b) tests in a 27-year old man.** Dashed lines mark the rise phase (a) or sit-to-stand transition (b) of the movement. In stair ascent, two steps are visualized. In sit-to-stand, one transition from sit to stand is visualized.

Fig 1 visualizes the vertical velocity, position and power during the rise phase in SA and the sit-to-stand transition in STS.

Duration-based estimations of power. Previous research has used power estimations for SA and STS based on performance duration. Because this approach has the highest potential for large-scale implementation, we additionally calculated power during unloaded SA and STS based on performance durations instead of accelerometry. SA power was estimated based on the following formula: SA power = ((body mass in kg) x 9.81m/s²) x (total stair height in m))/(total ascent duration in s) [31]. STS power was estimated using the following equation: STS power = ((thigh length in m + lower leg length in m–chair height in m) x body mass in kg x 9.81 m/s² x 5)/(total STS duration in s) [32].

#### Statistical analyses

Statistical analyses were performed using R software, version 1.0.153. The level of significance was set at p<0.05. All statistical procedures mentioned below have been reported in detail in previous papers of our group [7, 17].

Descriptive statistics were represented as means and standard deviations. For this purpose only, subjects were divided in three age categories (20–40 years, 40–55 years, 55–70 years). One-way analysis of covariance (ANCOVA) with Bonferroni post hoc tests were used to compare age categories. Body mass was used as a covariate.

To examine whether SA and STS performance are a good representation of LEP, Pearson’s correlation coefficients were calculated between SA or STS performance and LEP. To explore the relationship with parameters of duration, LEP was corrected for body mass.

To evaluate and compare average age-related changes (in % per year) in power and duration parameters (dependent variables), dependent variables were first log-transformed (natural log). A log-transformation of the dependent variable in regression models allows interpreting exponentiated β coefficients as percent changes [7, 17]. Linear regression analyses with the abovementioned log-transformed parameters as dependent variable and age as independent variable were performed. Body mass and sex were introduced as covariates in the regression analyses.

To study the effect of age and test method (i.e. leg extension, SA or STS) on power, linear mixed models were built using the function lmer provided by the R-package lme4. The dependent variable power was log-transformed to enable relative comparison of the age-related declines between test methods [17]. Age-by-test method in interaction was entered as fixed effect into the model. Subject was included in the model as random effect to correct for the repeated measures design. Sex and body mass and their possible interactions were analyzed in a series of separate linear mixed effects models using step-up model comparisons. This iterative model fitting procedure started with the basic model of investigating the effect of age, test method and age-by-test method on the dependent variable, then sequentially adding predictor terms. The Akaike information criterion (AIC) and a likelihood ratio test, performing the R-function ‘ANOVA’, were used to compare models. The final prediction model was visualized by means of a scatterplot with power (log-transformed) on the y-axis and age on the x-axis. Separate lines were plotted for leg extension, SA and STS. Similar linear mixed models analyses were performed to study the effect of the weighted vest on the age-related decline in SA and STS power.

## Results

Subject characteristics are described in Table 1. All SA and STS parameters were significantly related to LEP (p < 0.05) (Table 2). For both SA and STS, power parameters clearly show a better correlation with LEP than duration parameters (i.e. 0.80–0.86 for power and -0.41 –-0.66 for duration parameters, p < 0.05) (Table 2). The addition of a weighted vest (+10% body mass) during functional performance tests resulted in an increase in the relationship between LEP and duration parameters for the SA test, but not for the STS test.

**Table 1:** Subject characteristics and performance by age group. Data are presented as means ± SD.

				20–40 years	40–55 years	55–70 years	Age group difference*
			*n* (range)	35–38	28–29	28–31	
			Women (%)	52.6	48.3	48.4	
			Age (years)	29.28 ± 4.54	48.88 ± 3.94	62.02 ± 3.96	
			Body mass (kg)	72.65 ± 12.11	74.82 ± 12.27	73.04 ± 13.91	/
			Body height (cm)	174.92 ± 8.46	173.79 ± 10.30	169.21 ± 9.23	/
			BMI (kg/m²)	23.63 ± 3.21	24.59 ± 2.38	25.11 ± 3.65	/
Leg extension			Power (watt)	992.52 ± 261.56	882.60 ± 281.64	764.16 ± 244.01	a > bc
Stair ascent	Sensor-based analyses	unloaded	Total ascent duration (s)	1.45 ± 0.19	1.52 ± 0.17	1.78 ± 0.34	a > c
			Ascent rise duration (s)	1.25 ± 0.15	1.32 ± 0.16	1.56 ± 0.32	a > c
			Power (watt)	1136.01 ± 332.77	958.23 ± 305.51	738.83 ± 288.12	a > bc; b > c
	Duration-based estimation		Power (watt)	538.39 ± 109.36	526.64 ± 108.81	443.24 ± 117.88	ab > c
	Sensor-based analyses	loaded (+10%)	Total ascent duration (s)	1.48 ± 0.18	1.57 ± 0.17	1.81 ± 0.29	ab > c
			Ascent rise duration (s)	1.32 ± 0.15	1.41 ± 0.16	1.66 ± 0.33	ab > c
			Power (watt)	1089.73 ± 309.73	927.68 ± 321.94	736.18 ± 280.03	a > bc; b > c
	Duration-based estimation		Power (watt)	581.16 ± 121.70	560.15 ± 114.33	478.58 ± 129.31	ab > c
Sit-to-stand	Sensor-based analyses	unloaded	Total STS duration (s)	7.86 ± 0.71	8.47 ± 0.99	8.59 ± 1.01	a > bc
			Sit-to-stand transition duration (s)	4.10 ± 0.37	4.39 ± 0.49	4.47 ± 0.49	/
			Power (watt)	335.00 ± 92.21	344.60 ± 113.85	293.55 ± 81.84	ab > c
	Duration-based estimation		Power (watt)	182.21 ± 45.46	171.17 ± 53.3	158.45 ± 46.90	a > c
	Sensor-based analyses	loaded (+10%)	Total STS duration (s)	7.77 ± 0.80	8.31 ± 1.01	8.52 ± 1.04	a > c
			Sit-to-stand transition duration (s)	4.04 ± 0.45	4.27 ± 0.65	4.43 ± 0.47	a > c
			Power (watt)	388.80 ± 100.67	367.82 ± 109.82	340.61 ± 93.70	a > c
	Duration-based estimation		Power (watt)	203.96 ± 54.39	191.99 ± 59.90	175.05 ± 51.61	a > c

*Results of ANCOVA and Bonferroni post hoc with age as independent variable and body mass as covariate. a = age group 20–40 years, b = age group 40–55 years, c = age group 55–70 years

**Table 2:** Pearson correlation coefficients between stair ascent and sit-to-stand parameters and a standardized measurement of multi-joint leg-extensor power in men and women aged 20–70 years (N = 91–95).

				Leg-extensor power
Stair ascent	Sensor-based analyses	unloaded	Total ascent duration	-0.53
			Ascent rise duration	-0.50
			Power (watt)	0.80
	Duration-based estimation		Power (watt)	0.80
	Sensor-based analyses	loaded (+10%)	Total ascent duration	-0.66
			Ascent rise duration	-0.62
			Power	0.80
	Duration-based estimation		Power (watt)	0.88
Sit-to-stand	Sensor-based analyses	unloaded	Total STS duration	-0.44
			Sit-to-stand transition duration	-0.50
			Power	0.79
	Duration-based estimation		Power (watt)	0.86
	Sensor-based analyses	loaded (+10%)	Total STS duration	-0.42
			Sit-to-stand transition duration	-0.41
			Power	0.81
	Duration-based estimation		Power (watt)	0.85

All correlation coefficients are significant at P < 0.05. For correlation analyses with duration parameters, leg-extensor power (in watt) was divided by BM.

Results of linear regression models with log-transformed power and duration parameters as dependent variables and age, body mass and sex as predictor variables are presented in Table 3. No age by sex interaction effect was found, so this variable was not included in the models. The set of predictor variables was able to explain 67%-77% of the variance in power (i.e. in leg extension, SA and STS), but only 9%-42% of the variance in duration parameters (i.e. in SA and STS). Age was able to predict 25%-35% of sensor-based SA performance, 13% of duration-based SA power and 14% of LEP. Age was not a significant predictor of sensor-based STS power and only a limited predictor (4%) of duration-based STS power. The average annual age-related percent change was the highest for sensor-based SA power (-1.38%) and the lowest for total STS duration (+0.26%).

**Table 3:** Results of linear regression models with (1) age, sex and body mass as independent variables or with (2) age alone as independent variable. All dependent variables (power and duration) were log transformed. Exponentiated regression coefficients of the variable age represent annual age-related change (in %).

				Annual age-related change (in %) Model 1	Adjusted R² Model 1	Adjusted R² Model 2
Leg extension			Power (watt)	-0.86	0.77	0.14
Stair ascent	Sensor-based analyses	unloaded	Total ascent duration (s)	0.63	0.32	0.30
			Ascent rise duration (s)	0.66	0.36	0.34
			Power (watt)	-1.38	0.67	0.29
	Duration-based estimation		Power (watt)	-0.63	0.68	0.14
	Sensor-based analyses	loaded (+10%)	Total ascent duration (s)	0.60	0.42	0.34
			Ascent rise duration (s)	0.66	0.42	0.35
			Power (watt)	-1.28	0.69	0.25
	Duration-based estimation		Power (watt)	-0.61	0.77	0.13
Sit-to-stand	Sensor-based analyses	unloaded	Total STS duration	0.25	0.10	0.09
			Sit-to-stand transition duration (s)	0.27	0.18	0.11
			Power (watt)	-0.38	0.71	0.02 (n.s.)
	Duration-based estimation		Power (watt)	-0.42	0.75	0.04
	Sensor-based analyses	loaded (+10%)	Total STS duration	0.26	0.09	0.09
			Sit-to-stand transition duration (s)	0.29	0.10	0.08
			Power (watt)	-0.42	0.74	0.04
	Duration-based estimation		Power (watt)	-0.45	0.75	0.04

P < 0.05, n.s. = not significant

**Fig 2:**
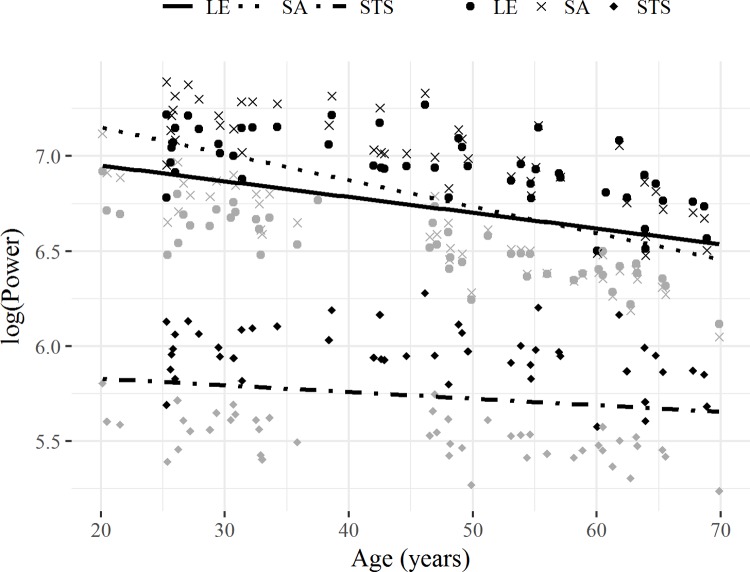
Measurements of leg-extensor (LE), sensor-based stair ascent (SA) and sensor-based sit-to-stand (STS) power in healthy adults (N = 92–96, age 20–70 years). The overall average age-related decline is represented by a solid linear fit for leg-extension, a dotted linear fit for stair ascent and a dot-dashed linear fit for sit-to-stand. Men are presented in black, women in grey. Data are displayed on a log scale.

Linear mixed effects models demonstrated that the age-related decline in sensor-based SA power exceeds the decline in LEP (p < 0.001), whereas sensor-based STS power declines less than LEP (p = 0.002) (Fig 2 and Table 4). Separate linear mixed effects models did not show a significant effect of load on the age-related decline in sensor-based SA and STS performance (p > 0.05). Participants developed on average more power in the loaded condition for STS and in the unloaded condition for SA (p < 0.05).

**Table 4:** Results of linear mixed effects model with power (log-transformed) as independent variable and age, method (i.e. leg extension, stair ascent, sit-to-stand), sex and body mass as fixed factor, age and test method in interaction and subject as random factor. Leg extension (for method) and men (for sex) were used as the reference.

Factor	Estimate	SE	p-value
Intercept	6.001	0.095	< 0.001
Age	-0.009	0.001	< 0.001
Method stair ascent	0.200	0.045	< 0.001
Method sit-to-stand	-1.115	0.044	< 0.001
Sex	-0.252	0.034	< 0.001
Body mass	0.011	0.001	< 0.001
Age*Method stair ascent	-0.005	0.002	< 0.001
Age*Method sit-to-stand	0.005	0.002	0.002

SE = standard error,

* refers to an interaction term.

## Discussion

Leg-extensor power has been found to be a crucial contributor to physical functioning in older adults [14]. Consequently, the evaluation of LEP has been suggested as a sensitive method for the detection of early age-related deterioration in function, but large-scale applicability of traditional methodologies is limited. The vertical power required to elevate the body’s center of mass during stair ascent or a transfer from sit to stand can be estimated using sensor-based trunk kinematics, but its relationship to LEP and its sensitivity to early age-related changes remained to be investigated, which were the primary aims of the current study. In addition, the effect of a 10%-load during SA and STS tests on the sensitivity to detect early age-related changes was examined. The main findings were: (1) SA and STS power are highly related to LEP and substantially more related as opposed to duration parameters, (2) the age-related decline detected in SA power exceeds the decline in LEP and STS power, suggesting that SA is more sensitive to detect early age-related changes, (3) the addition of 10% load during SA and STS did not influence the detected age-related decline.

Biomechanical analyses have shown that the knee- and hip-extensor muscles produce the greatest concentric power output during standing from a chair [33]. Similarly, the knee extensor muscles play a dominant role in the progression from one step to the next in stair climbing, together with the ankle plantar flexors [34, 35]. The importance of knee-extensor strength and power in SA and STS have already been established in previous research [31, 36–39]. It is therefore not surprising that, in the current study, both SA and STS performance were related to multi-joint LEP. Interestingly, sensor-based power parameters (r = 0.80–0.81) were clearly more associated with LEP than duration parameters (r = -0.41 –-0.66) in both SA and STS tests, which stresses the added value of power parameters in functional tests. It should be noted that previous research has used other simple alternative ways to estimate power in SA and STS tests, for example with smartphone applications [40, 41], Tendo Weightlifting Analyzer [42], or by only using a stopwatch [31, 32]. Because stopwatch-based approaches have the highest potential for large-scale implementation, we additionally calculated power during SA and STS based on performance durations instead of accelerometry. These duration-based power estimations were positively correlated to LEP and to a similar extent as our sensor-based analysis (i.e. r = 0.80 for SA and r = 0.86 forSTS). Both sensor- and duration-based power estimations are therefore valuable to evaluate LEP in clinical settings.

The use of inertial measurement units to distinguish different phases of a functional movement is becoming more common. It allows for a more detailed analysis of sub-durations of the movement, such as the sit-to-stand and stand-to-sit transition in STS [29, 43]. As total durations might be influenced by phases where no concentric power (i.e. stand-to-sit transition in STS) or limited power is produced, we additionally calculated sub-durations for the rise (in SA) or sit-to-stand transition phase (in STS). However, these sub-durations appeared to have no added value over total durations to evaluate LEP in a healthy well-functioning population.

The evaluation of LEP has been suggested as sensitive screening method for early age-related deterioration of neuromuscular function [7], therefore we investigated the effect of age on power. LEP declined at an average rate of -0.86% per year between the ages of 20 to70 years, which is in line with previous reports on leg-extensor power decline over a similar age range in single joint [7, 17] and multi-joint [17, 44–46] tests. To the authors’ knowledge, no research has investigated the age-related decline in SA power over the adult life span. The current study showed an average decline in sensor-based SA power of -1.38% per year, which exceeded the decline in LEP. As previously shown, successful stair performance is highly dependent upon both knee and angle joint power [34, 47, 48]. During the ageing process, a decline in the neuromuscular performance of plantar and dorsi-flexor muscles is commonly observed [49], which might explain the greater age-related decline in SA power as opposed to LEP, where ankle movements were limited. In addition, SA includes a balance component and therefore evaluates LEP from a more functional perspective than standardized LEP measurements do. As opposed to SA power, we found a significantly lower average age-related decline rate (-0.38% per year) for sensor-based STS power. This might be a result of a ceiling effect in STS power, as visualized in Fig 2 and Table 1, where no clear differences were observed between the age categories of 20–40 years and 40–55 years. As confirmed in the linear regression models in Table 3, age is not a good predictor of STS performance in healthy well-functioning adults from 20 to 70 years of age. This might seem contradictory to the finding that STS power is highly related to LEP. However, this can partly be attributed to the major contribution of body mass to both power measurements. Interestingly, age is able to explain 29% in sensor-based SA power, which is markedly higher than the predictive value of age in LEP (14%) or sensor-based STS power (2%, not significant). Age was less of a contributor to duration-based SA power and age-related declines were less pronounced (-0.63%/year), underlining the added value of the sensor-based analysis of power. Overall, the results stress the potential of the stair ascent test for detection of functional and neuromuscular decline in ageing and suggests that stair ascent might be even more sensitive to age-related changes than LEP.

The addition of 10% load during SA and STS did not influence the detected age-related decline of the power component nor the relationship with LEP. More specifically for STS, power production was significantly higher in the loaded compared to the unloaded condition, indicating that the higher load did not induce a marked decline in vertical acceleration. In other words, both the unloaded and loaded condition in STS were clearly not challenging enough, considering that the addition of 10% load did not reduce performance. This amount of load was not able to overcome the ceiling effect, resulting in limited age-related differences. Higher loads or other strategies, such as the lowering of seat height [50] or allowing jumping [22], should be considered to overcome the ceiling effect in STS tests in well-functioning adults. For SA, power production decreased when adding a 10% load. This means that vertical acceleration was reduced because of the load, pointing out that the loaded test was probably more challenging. Nevertheless, given that the unloaded condition was already challenging enough to induce marked age-related declines, the loaded condition did not add value to the age-related comparison.

A limitation of the current study is its cross-sectional design; magnitudes of decline may differ in longitudinal comparisons. Although we included a large age range (20–70 years), sample size is limited and does not allow for interpretation of patterns of decline, such as the starting point of decline or accelerated decline rates after a certain age. Therefore, we were only able to calculate average linear age-related declines, which is an oversimplification of the real-life situation and inevitably over- and underestimates the magnitude of the decline in young and older adults respectively. However, the age-related decline rate in LEP was similar to the decline in a previous large-scale study [7]. In addition, correlation coefficients were calculated over the full age range in the current study, which might have overestimated the relationship, considering that high performing (i.e. young) and low performing (i.e. old) subjects are combined in one analysis. Therefore, we additionally calculated correlation coefficients for subjects ≥ 50 years (N = 38). Results did not change, with sensor-based SA and STS power being more related to LEP (r = 0.77 and r = 0.85 respectively) than duration parameters (r = -0.34 for SA, r = -0.43 for STS).

## Conclusions

The current study emphasized the potential of SA to detect deterioration in neuromuscular function. SA and STS power are highly related to LEP and substantially more as opposed to commonly used duration parameters. The average age-related decline was markedly higher in sensor-based SA power (-1.38% per year) than in both LEP (-0.86% per year) and sensor-based STS power (-0.38% per year). It appears that SA provides a more complete picture of neuromuscular decline by including a balance component and plantar- and dorsi-flexor activity compared to traditional leg-extensor measurements. This suggests that SA is more sensitive to detect age-related changes than LEP. On the contrary, STS shows a ceiling effect in well-functioning adults and is therefore less sensitive to age-related changes. The addition of 10% load during STS was not able to overcome that ceiling effect, suggesting that other strategies should be used to improve its sensitivity. Future research should additionally investigate the sensitivity of sensor-based stair ascent to detect changes in power in response to training.
